# Dormant season grazing on northern mixed grass prairie agroecosystems: Does protein supplement intake, cow age, weight and body condition impact beef cattle resource use and residual vegetation cover?

**DOI:** 10.1371/journal.pone.0240629

**Published:** 2020-10-13

**Authors:** Samuel A. Wyffels, Darrin L. Boss, Bok F. Sowell, Timothy DelCurto, Janice G. P. Bowman, Lance B. McNew

**Affiliations:** 1 Northern Ag Research Center, Montana State University, Havre, Montana, United States of America; 2 Department of Animal and Range Sciences, Montana State University, Bozeman, Montana, United States of America; University of Illinois, UNITED STATES

## Abstract

Dormant season livestock grazing reduces reliance on harvested feeds, but typically requires protein supplementation to maintain animal performance. Individual variation in supplement intake can impact animal performance; however, it is unknown if this variation leads to individual or herd-level effects on grazing behavior, resource utilization, and grazing impacts to native rangelands. To examine effects of protein supplementation on dormant season cattle resource use and, subsequently, post-grazing habitat conditions, we examined cattle grazing behavior, resource utilization and biomass removal of vegetation on a native rangeland in Montana. A commercial herd of 272 (yr 1) and 302 (yr 2) cows grazed a 329-ha rangeland pasture from November to January. Intake of a 30% crude protein supplement was measured for each individual. Five individuals within each of six age groups were equipped with GPS collars. Time spent grazing declined with supplement intake ($\hat{\beta }$ = −0.05 ± 0.02; *P* < 0.01). Distance traveled per day had a positive asymptotic association with supplement intake ($\hat{\beta }$ = 0.35 ± 0.09; *P* < 0.01). On average, resource utilization by cattle grazing dormant season forage decreased with terrain ruggedness ($\hat{\beta }$ = −0.09 ± 0.03), but was unrelated to aspect, temperature and wind speed. Notably, we observed high individual variability in resource utilization for elevation, distance from supplement and water. A post-hoc analysis suggested that individual attributes (age, body weight, supplement intake) influenced cattle resource use. At moderate stocking rates, dormant season livestock grazing did not affect residual vegetation conditions (*P* values > 0.22). However, residual cover of forbs and litter increased with relative grazing intensity ($\hat{\beta }$ = 1.04 ± 0.41; $\hat{\beta }$ = 3.06 ± 0.89; *P* ≤ 0.05). In summary, high individual variability in grazing resource utilization of cattle suggests individual-level factors could be the dominant drivers in grazing behavior and landscape use.

## Introduction

Economic efficiency of cattle production is threatened by high feed and input costs [1]. To improve profitability and transition to reduced reliance on transported harvested feeds, many cow-calf producers have adopted management strategies involving dormant season grazing [2]. The primary goal in a forage-based livestock production system is to obtain optimal animal performance while effectively utilizing the forage resource base. Western U.S. livestock producers primarily manage for spring calving cows, weaning calves in the fall. As a result, most cattle enter the winter dormant grazing season without the added nutritional requirements associated with lactation [3]. Dormant range forage is deficient in nutrients and may result in an inability to meet production expectations [4–6]. Providing protein supplements to grazing beef cattle during times of low forage quality can improve animal performance and provide increased economic returns [4, 7, 8]. However, supplementation strategies assume that all animals consume a targeted quantity of supplement and deviation from the target can have strong effects on animal nutirent status [9].

The spatial component of herbivory is a central aspect of domestic livestock ecosystems but has remained difficult to interpret [10]. Mechanisms that influence grazing distribution can be classified into two factors: exogenous, the physical environment in which livestock graze, such as topography, thermal environments and forage resources [11–14], and endogenous characteristics such as social learning, spatial memory, age, experience, body weight and condition [13, 15–17]. Thus, cattle grazing the same pasture under the same environmental conditions can have very different grazing patterns [18]. Supplementation alters the nutrient status of grazing livestock, which can also have strong influences on individual grazing behavior [17, 19]. The act of supplementation alone can change grazing distribution on rangelands and daily grazing activities, altering the distribution of vegetation use based on location of supplements [20–22]. Therefore, it is likely that grazing behavior may vary with individual animal protein supplement intake, age, body weight and condition in dormant season grazing systems. However, interactions of exogenous factors with endogenous attributes on grazing behavior are less understood [15].

Grazing behavior of livestock plays a key role in grassland ecosystem function having both direct and indirect influences on vegetative communities [23–25]. Cattle alter their grazing behavior in response to nutritional factors, where providing a protein supplement often increases the intake of dormant forage and alters grazing distribution [5, 26, 27]. Vegetation conditions of western rangelands are inherently heterogeneous [28–30], thus, protein supplementation has been used as a management tool to increase use of dormant forage and promote uniform utilization across the pasture [26]. Although, dormant forage is typically tolerant of grazing pressure [31], grazing management that promotes uniform utilization can result in the homogenization of vegetation conditions and an overall decline in ecosystem structure, function and biodiversity [23, 32, 33]. Thus, it has been proposed that heterogeneity of vegetation structure, composition, and biomass should be the foundation of conservation and ecosystem management [34–36]. Despite grazing livestock’s keystone role as grassland ecosystem engineers [23], we currently have little understanding of how protein supplementation and endogenous attributes of grazing cattle interact with exogenous properties of the pasture in determining cattle use and the corresponding effects on residual vegetation cover, structure and heterogeneity.

Information relating supplement intake, cow age, body weight and condition to individual grazing distribution and behavior is lacking. Therefore, the intent of this study was to evaluate (1) the influence of supplement intake, age, body weight and condition on grazing activity (time spent grazing and distance traveled per day) and resource utilization by cattle, and (2) the influence of dormant season use on residual vegetation cover, structure and heterogeneity. We expect that environmental factors and individual animal attributes have multi-faceted effects on distribution by supplemented cattle grazing dormant rangeland in northern mixed-grass prairies. We hypothesized that cattle use is affected by endogenous attributes of the animal and distribution of use will have strong effects on vegetation structure. System-level impacts are likely mediated by the provision of supplement, as well as, uncontrolled environmental conditions.

## Materials and methods

This study was conducted at the Thackeray Ranch (48° 21' N 109° 30' W), part of the Montana Agricultural Experiment Station located 21-km south of Havre, MT. Climate is characterized as semi-arid steppe with an average annual precipitation of 410-mm. Vegetation is dominated by Kentucky bluegrass (*Poa pratensis L*), bluebunch wheatgrass (*Pseudoregnaria spicata* [Pursh] A. Love), and rough fescue (*Festuca scabrella Torr*). Preciptiation was higher in the winter of 2017 – 2018 than 2016 – 2017 (4.06, 2.90 cm), however, temperatures durring the 2016 – 2017 winter were substantially cooler than the winter of 2017 – 2018 (−9.60, −2.90°C), resulting in higher amounts and prolonged periods of snow in the first year of the study (S1 Table).

A commercial herd of bred cows (Angus, Angus × Simmental) ranging in age from 1- to 12-years-old were grazed on a 329-ha rangeland pasture (~1.5 AUM ha^-1^) during 2 years (272 cows in the 1st year, and 302 cows in the 2nd year). The winter grazing season occurred from December 1, 2016 to January 12, 2017, and November 1, 2017 to December 31, 2017. The rangeland pasture used in our study was left un-grazed during the growing season to stockpile available forage for winter grazing, typical of winter grazing management in the Pacific Northwest and Northern Great Plains. A perennial stream runs through the center of the pasture and is used for livestock watering. All cattle had free-choice access to a 30% crude protein (CP) self-fed canola meal-based pelleted supplement with a standard 25% salt to limit over consumption (S2 Table). The target daily intake was 0.91-kg ∙ cow^-1^. Supplement feeders were located centrally within the pasture. All cattle were weighed, and body condition scored at the initiation and completion of the research trial (S3 Table). The use of animals in this study was approved by the Agricultural Animal Care and Use Committee of Montana State University (#2015-AA04). Cattle were maintained in production upon completion of this study, no animals were euthanized.

### Sampling

We established seventy-five 30-m transects randomly within the study pasture. Vegetation production, canopy cover and visual obstruction readings (VOR) were measured at six 0.1-m^2^ plots located every five meters along each transect. We estimated canopy cover of plant functional groups (grass, forb, shrub), cover of bare ground and litter at each plot using the six-cover class Daubenmire method [37]. Ground cover of plant functional groups, bare ground and litter influence the abundance and demography of grassland bird species that serve as indicators of grassland ecosystem health [23, 38, 39]. We measured visual obstruction readings in four cardinal directions using a 1-meter Robel pole [40]. Visual obstruction represents a measure of the vertical structure and density of vegetation and is typically correlated with aboveground biomass [40, 41]. All measurements were taken pre- and post-grazing to evaluate the effects of relative grazing intensity on residual vegetation cover and structure across the pastures. All pre- and post-grazing vegetation sampling were conducted within a 10-day period just prior to the initiation of grazing and at the end of the study period. We estimated pre-grazing vegetation functional group production using the dry weight rank method and clipping each plot [42, 43]. Clipped samples were placed in a forced air oven at 60°C for 48 hours and then weighed. Pre-grazing vegetation samples were composited by transect and ground to pass through a 1-mm screen in a Wiley mill and analyzed in duplicate for nitrogen (Leco CN-2000; Leco Corporation, St. Joseph, MI), and fiber (NDF and ADF; Ankom 200 Fiber Analyzer, Ankom Co., Fairport, NY; Table 1).

**Table 1:** Average annual grass production (± SE, kg/ha), Crude Protein (CP ± SE; %), Neutral Detergent Fiber (NDF ± SE; %) and Acid Detergent Fiber (ADF ± SE; %) of the experimental paddock for the 2 years of grazing (2016 – 2017, 2017 – 2018) at the Northern Agricultural Research Center Thackeray ranch, Havre, MT.

	Grass Production (kg/ha)	CP (%)	NDF (%)	ADF (%)
**Year 1**	3128.03 ± 21.78	6.85 ± 0.03	70.46 ± 0.08	43.92 ± 0.05
**Year 2**	2709.42 ± 23.71	7.07 ± 0.03	70.09 ± 0.08	44.46 ± 0.05

Grazing activity was monitored with Lotek GPS collars (3300LR; Lotek Engineering, Newmarket, Ontario, Canada) containing head position sensors that record daily space use, as well as, timing and location of grazing activities [44–46]. All cattle were assigned to one of six age classifications (1-yr-old, 2 & 3-yr-olds, 4 & 5-yr-olds, 6 & 7-yr-olds, 8 & 9-yr-olds, and ≥ 10-yr-old) and randomly selected for GPS collars (5 collars per age class). Age classifications were based on previous research examining the influence of age on grazing behavior and distribution patterns of cattle within a large mixed-conifer allotment pasture [15]. Each individual animal was equipped with an electronic ID tag (Allflex USA, Inc., Dallas-Ft. Worth, TX) attached to the left ear for the measurement of individual supplement intake using a SmartFeed Pro self-feeder system (C-Lock Inc., Rapid City, SD) which provided a total of 8 feeding stations. Each collar was configured to record GPS positions at 15-minute intervals, and head position, vertical/horizontal movements at 5-minute intervals. We then separated grazing from non-grazing activities using the binary classification methods developed by Augustine and Derner [47] to examine time spent grazing and cattle foraging distribution. By limiting observations to grazing locations we were able to determine important foraging areas rather than general pasture occupancy [15].

An Onset HOBO U30-NRC Weather Station (Bourne, MA, USA) was placed near the supplement feeders and programmed to collect air temperature, relative humidity, and wind speed and direction data every 15 min for the entirety of the grazing period. We predicted fine scale wind speed (30-m^2^ resolution) across all pastures using average daily wind measurements collected on site, ArcGIS spatial analyst tool, a digital elevation model at 30-m^2^ resolution, and WindNinja wind prediction software [48]. In addition, we deployed HOBO Pendant® Temperature/Light Data Logger (Onset Computer Corporation, Bourne, MA) at each randomly selected transect location within the pasture that were programmed to collect fine-scale ambient temperature every 30-minutes. We modeled the effects of physical properties (e.g., aspect, elevation and slope) on fine-scale temperature of the pastures using generalized linear models. Model results were used to create spatially explicit predictions of average temperature and wind conditions across the experimental pasture at a 30-m^2^ resolution, which were used as covariates in subsequent resource utilization modeling.

All supplementation and water locations within the study pasture were located via handheld GPS (spatial error < 10-m). Using the spatial analysis tool in ArcGIS (Environmental Systems Research Institute, Redlands, CA) and a digital elevation model at 30-m^2^ resolution [49], we created additional spatial covariate layers representing aspect, solar radiation, terrain ruggedness [the sum change in elevation between a grid cell and its eight neighboring cells; 50] and horizontal distance from supplement locations and water sources at 30-m^2^ resolution.

### Statistical analysis

Time spent grazing and distance traveled per day were estimated daily for individual GPS collared animals and daily supplement intake was measured for all animals (S1 and S2 Appendices). We evaluated the effects of cow age, supplement intake, body condition and weight on time spent grazing and distance traveled with generalized linear mixed models using individual animal as a random intercept. We hypothesized that individual animal attributes could elicit one of three behavioral responses (linear, pseudothreshold, quadratic). Variables hypothesized to exhibit a pseudothreshold pattern were tested with asymptotic models by evaluating the natural log of the explanatory variable [ln[x + 0.001]; 51]. We used Akaike’s Information Criterion adjusted for small sample sizes (AIC_c_) to evaluate support for competing models reflecting hypotheses about the effects of individual animal attributes on time spent grazing and distance traveled by cattle [52]. Models with ΔAIC_c_ ≤ 2 that differed from the top model by a single parameter were excluded if confidence intervals of parameter estimates overlapped 0 [ie., were non-informative; 53]. When multiple models were supported, we used model-averaged estimates of beta-coefficients ["MuMIn” package for R; 54]. Model fit was then evaluated by calculating marginal and conditional r^2^ values for generalized linear mixed models [55].

To model relative resource selection during the dormant season, individual GPS-collared cows were defined as the biological unit of interest. We used multiple regression in a resource utilization function (RUF) analysis to relate individual cow space use, quantified as a continuous and probabilistic variable, to pasture level covariates [RUF; 56, 57]. Resource utilization functions increase sensitivity for detecting resource selection and reduce errors associated with location estimation by quantifying inter-animal variation in resource use and independently incorporating an individual’s entire distribution of use while accounting for spatial autocorrelation of grazing locations [56–58].

Due to the pasture management unit defining the home range of grazing cattle, GPS data were used to build RUFs quantifying animal selection of environmental and vegetation conditions within the pasture [third-order scale; 59]. We created a raster representing the specific utilization density distribution for the grazing locations of each individual in the pasture using Geospatial Modeling Environment [60]. Relative use values were bound between 1 and 99, for each 30 m^2^ cell based off of the relative volume of utilization distribution in that cell [57]. Environmental covariates expected to influence resource utilization included temperature, wind, solar radiation, distance to supplement and water (horizontal), elevation, terrain ruggedness and aspect, annual forage production and chemical composition. Using the ‘raster’ function in R, environmental covariate and individual relative use rasters were stacked and converted to spatially explicit data files as input for the ruf.fit package [S3 Appendix; 58]. Prior to modeling, individual relative use values were log-transformed to meet the assumptions of multiple regression models. Resource utilization functions with standardized β coefficients were generated using the ruf.fit package in R and evaluated for each individual to represent the influence of the environmental covariates on cattle resource utilization [57, 61].

Herd level inferences were developed by calculating the mean standardized β coefficients ($\hat{\bar{\beta }}$) and variance that incorporated individual animal variation for each environmental factor [57]. Standardized coefficients with 95% confidence intervals that do not overlap zero were considered significant predictors of resource use [56, 57]. If a resource utilization coefficient was significantly different from zero, we inferred that resource use was greater or less than expected based on availability of the resource within the experimental pasture [56, 57]. For environmental factors eliciting high herd-level variability in habitat selection (herd level SE of standardized coefficients > 0.25), we conducted a post hoc analysis evaluating the effects of cow age, supplement intake, body weight and condition on resource use coefficients relative to each habitat covariate using generalized linear models (S4 Appendix). We investigated three behavioral responses (linear, pseudothreshold, quadratic) that individual animal attributes may have on resource selection coefficients. We used Akaike’s Information Criterion adjusted for small sample sizes [AICc, 52] to evaluate support for competing models reflecting hypotheses about the effects of animal attributes on resource use by cattle. Model fit was then evaluated by calculating a multiple r^2^ value for generalized linear models.

Geo-referenced rasters representing estimates of vegetation composition, production and chemical composition were not available at the appropriate spatial resolution to incorporate in the RUF analysis. Therefore, to evaluate the relationship of vegetation characteristics and resource use of grazing cattle, we extracted the relative resource use value for each individual at each of the transect location within the pasture and paired it with the corresponding vegetation measurements (S5 Appendix). We evaluated the effects of vegetation (e.g., production, composition and chemical composition) and cow age, body condition, and supplement intake (linear, pseudothreshold, or quadratic response) on relative use with generalized linear mixed models using individual animal as a random effect. To avoid overfitting our resource use models, we conducted a preliminary multicollinearity analysis to select uncorrelated (|r| < 0.6) variables that are ecologically relevant and feasible to measure [62]. If covariates were correlated, we fitted preliminary resource utilization models and evaluated relative support of each individual variable using AIC_c_ [52]; we retained the variable with more relative support for further modeling and discarded the correlated variables [63]. Support for competing models reflecting hypotheses about the effects of various vegetation and individual animal attributes on relative use by cattle were evaluated using AIC_c_ [52]. Model fit was then evaluated by calculating marginal and conditional r^2^ values for generalized linear mixed models [55].

To evaluate the relative effects of grazing intensity on the residual cover of vegetation functional groups and visual obstruction, we calculated the overall density of grazing locations within a 50-m radius of each transect location for both years. We then calculated the relative difference in mean transect level vegetation cover and visual obstruction from pre- to post-grazing for each year. Patch level heterogeneity of vegetation cover and visual obstruction was calculated by subtracting the pre-grazing transect level standard deviation from the post-grazing standard deviation for both years (S6 Appendix). We used an analysis of covariance (ANCOVA) with generalized linear models including year as a categorical variable, density of grazing locations (grazing intensity) as a continuous variable, and an interaction of grazing intensity by year to evaluate the effects of grazing intensity and year on residual vegetation cover, visual obstruction and patch level heterogeneity of residual vegetation cover and visual obstruction. Data were plotted and log-transformed if needed to satisfy assumptions of normality and homogeneity of variance. An alpha ≤ 0.05 was considered significant. Shrub cover as a functional group of vegetation was not analyzed as approximately 90% of the total shrub canopy cover was of deciduous species. Post-grazing samples were taken prior to leaf budding making it difficult to evaluate the effects of grazing on residual shrub canopy cover. All statistical analyses were performed using program R [64].

## Results

### Grazing behavior and resource use

#### Time spent grazing

**Fig 1:**
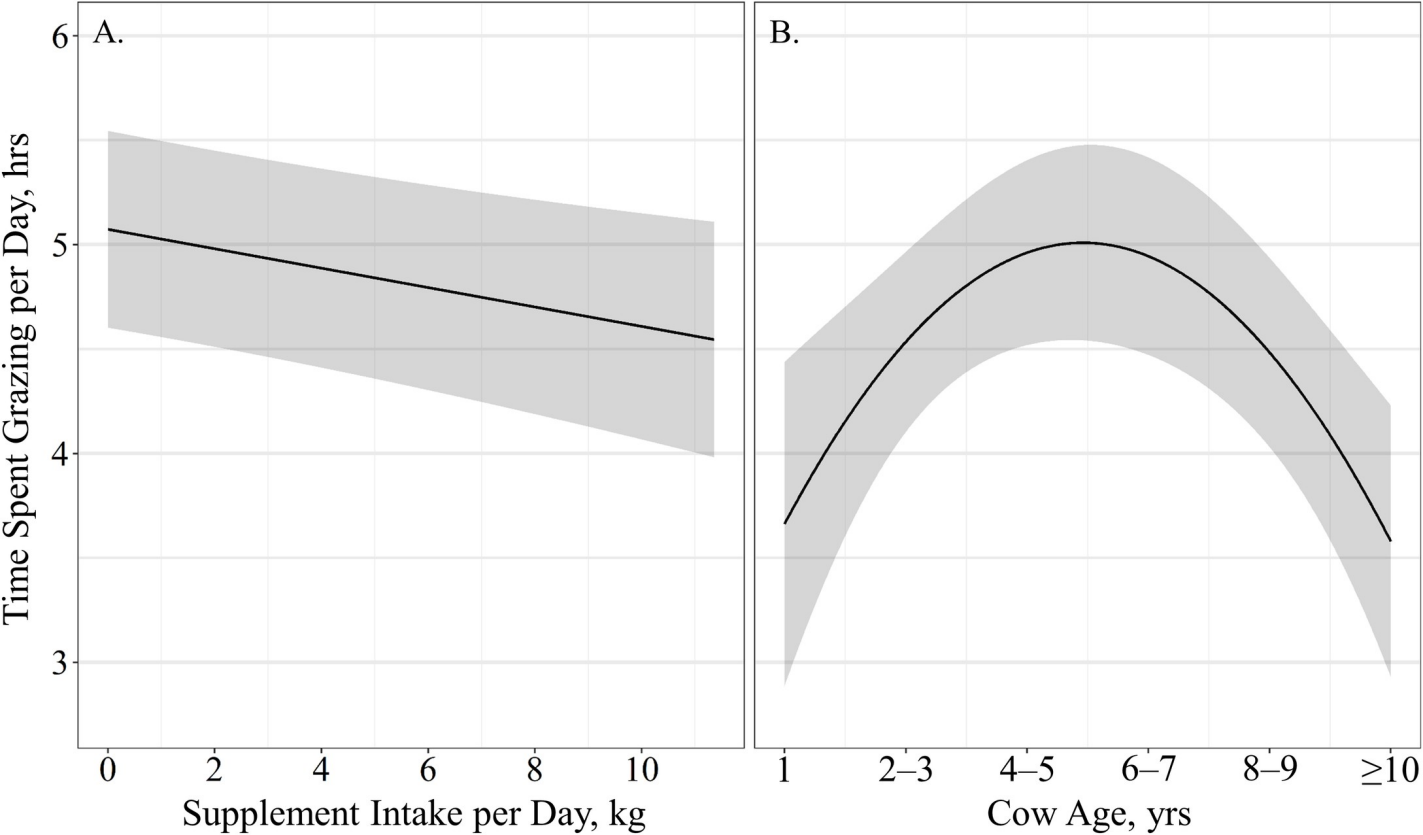
Predicted relationships (± 95%CI represented in the shaded area) between (**A**) average supplement intake per day (kg) and (**B**) cow age (years) on time spent grazing per day (hrs) by cattle grazing dormant northern mixed grass rangeland in 2016 – 2017 & 2017 – 2018 at the Northern Agricultural Research Center Thackeray ranch, Havre, MT.

The effects of cow age, supplement intake, and body condition and weight on time spent grazing per day and distance traveled per day were evaluated for 29 cows from December 1, 2016 to January 12, 2017 and 29 cows from November 1, 2017 to December 31, 2017. Models containing a quadratic effect of cow age received 61% of the support among candidate models for time spent grazing per day (Table 2). Models containing supplement intake, body condition, body weight and an interaction of cow body condition by cow weight were also supported, however, the parameter estimates for cow body condition ($\hat{\beta }$ = 52.74 ± 64.39; *P* = 0.41), body weight ($\hat{\beta }$ = 14.55 ± 17.11; *P* = 0.40) and the interaction of the cow body condition by cow weight ($\hat{\beta }$ = −8.09 ± 9.95; *P* = 0.42) may be non-informative as confidence intervals of the effect size overlap 0. Time spent grazing was negatively associated with supplement intake ($\hat{\beta }$ = −0.05 ± 0.02; *P* < 0.01), where time spent grazing per day decreased 3-min for every kg of supplement intake (Fig 1A). Time spent grazing was quadratically affected by cow age ($\hat{\beta }$_Age_ = 1.54 ± 0.46, $\hat{\beta }$_Age_^2^ = −0.22 ± 0.06; *P* < 0.01), indicating that cows maximized time spent grazing at ages of 4 – 7 years (Fig 1B). The top model containing all supported variables among candidate models had a conditional *r*^2^ of 0.51; however, the marginal *r*^2^ was only 0.12 suggesting age, supplement intake, body weight and body condition only account for 12% of the variation associated with time spent grazing.

**Table 2:** Model selection for models evaluating the effects of cow age, body condition and supplement intake on time spent grazing per day (hrs) and distance traveled per day (km) by cattle grazing dormant rangeland in 2016 – 2017 & 2017 – 2018 at the Northern Agricultural Research Center Thackeray ranch, Havre, MT.

Model^b^	K^c^	AICc^d^	ΔAICc^e^	*w*_*i*_^f^	*r*^2^m^g^	*r*^2^c^h^
Time Spent Grazing per Day						
Age^2^ + Supplement Intake +ln(Body Condition) × ln(Body Weight)	9	10113.50	0.00	0.28	0.12	0.51
Age^2^ + ln(Body Condition) × ln(Body Weight)	8	10113.53	0.03	0.28	0.11	0.51
ln(Body Condition) × ln(Body Weight)	6	10115.92	2.42	0.08	0.02	0.51
Constant (null)	3	10123.61	10.11	0.00		
Distance Traveled per Day						
ln(Age) + ln(Supplement Intake) × ln(Body Condition) + ln(Body Condition) × ln(Body Weight)	10	8643.60	0.00	0.40	0.18	0.26
ln(Age) + ln(Supplement Intake) × ln(Body Condition)	7	8646.05	2.45	0.12	0.18	0.25
ln(Age) + ln(Supplement Intake) + ln(Body Condition) × ln(Body Weight)	8	8646.16	2.56	0.11	0.18	0.26
ln(Age) × ln(Body Weight) + ln(Supplement Intake) × ln(Body Condition)	9	8646.29	2.68	0.11	0.18	0.26
ln(Age) + ln(Supplement Intake)	5	8647.31	3.71	0.06	0.17	0.25
ln(Age) + ln(Supplement Intake) × ln(Body Condition) + ln(Body Weight)	8	8647.37	3.77	0.06	0.18	0.26
Constant (null)	3	9027.40	383.80	0.00		

^a^Only models with Akaike weights (*w*_i_) ≥ 0.05 are presented except for the null model.

^b^Cow is used as a random variable in all models.

^c^K = number of parameters.

^d^Akaike’s information criterion adjusted for small sample size.

^e^Difference in Akaike’s information criterion adjusted for small sample size compared to the best model.

^f^Akaike weight.

^g^Marginal R^2^.

^h^Conditional R^2^.

#### Distance traveled

**Fig 2:**
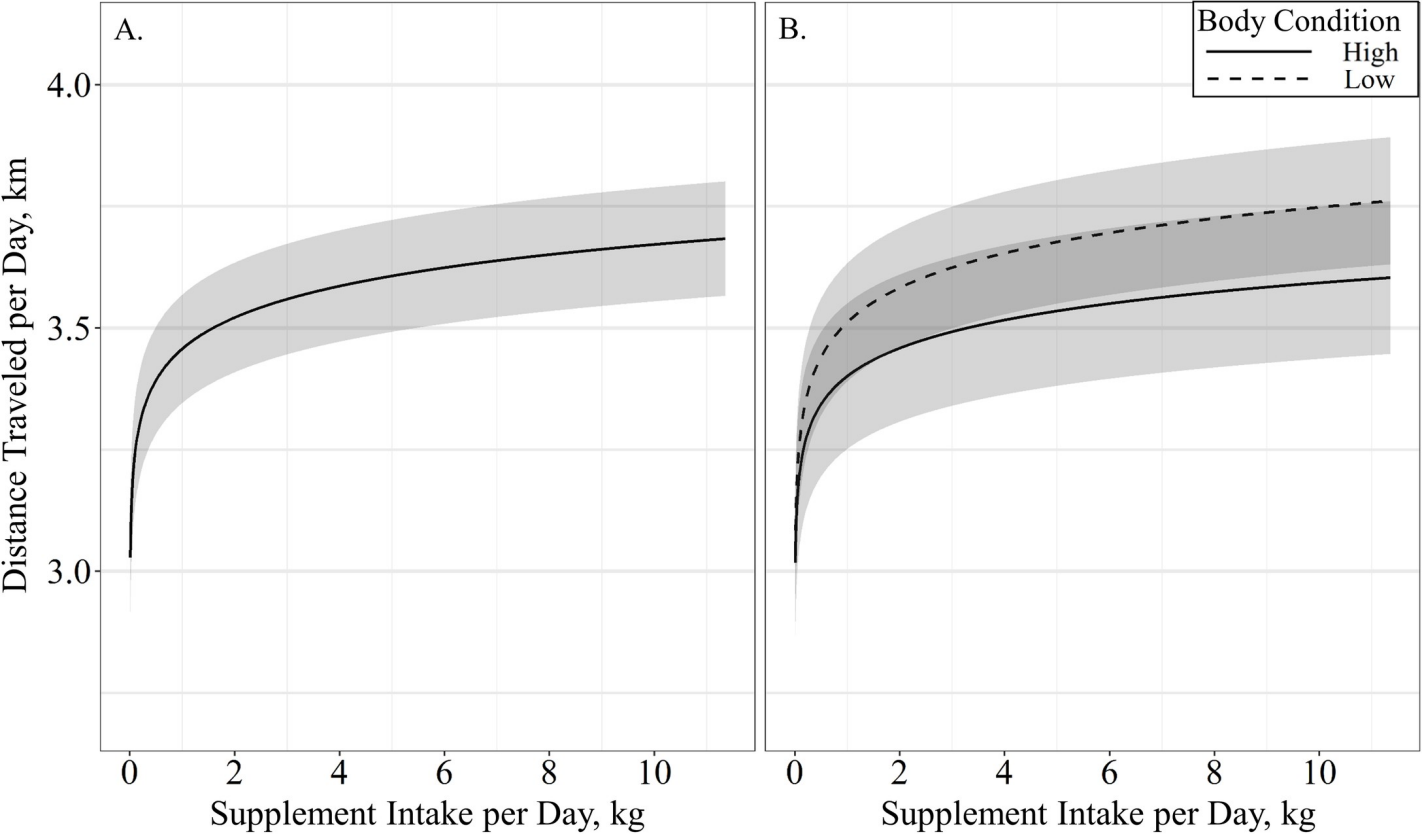
Predicted relationships (± 95%CI represented in the shaded area) between (**A**) average supplement intake per day (kg) and (**B**) the interaction of average supplement intake per day by body condition on distance traveled per day (km) by cattle grazing dormant northern mixed grass rangeland in 2016 – 2017 & 2017 – 2018 at the Northern Agricultural Research Center Thackeray ranch, Havre, MT.

A single top model containing age and supplement intake received 40% of the relative support of the data when determining the effects of cow age, supplement intake, body condition and weight on distance traveled per day (Table 2). Body condition, body weight and the interactions of body condition by supplement intake, age by body weight, and body condition by body weight were also supported, although the parameter estimates for age ($\hat{\beta }$ = −5.56 ± 4.18; *P* = 0.19), cow body condition ($\hat{\beta }$ = −16.12 ± 20.28; *P* = 0.43), body weight ($\hat{\beta }$ = −5.26 ± 5.30; *P* = 0.33) and the interactions of the cow body condition by body weight ($\hat{\beta }$ = 2.38 ± 3.14; *P* = 0.45) and cow age by body weight ($\hat{\beta }$ = 0.83 ± 0.66; *P* = 0.21) may be non-informative with confidence intervals of the effect overlapping 0. Distance traveled per day had an asymptotic association with supplement intake ($\hat{\beta }$ = 0.35 ± 0.09; *P* < 0.01), indicating a rapid increase in travel with supplement intake that begins to level at 2.5-kg and is maximized at 10-kg per day (Fig 2A). A supplement intake by body condition interaction was also supported ($\hat{\beta }$ = −0.15 ± 0.05; *P* < 0.01), where cow body condition had a larger effect on distance traveled per day at high levels of supplement intake (Fig 2B). The top model evaluating the effects on distance traveled had a conditional *r*^2^ of 0.26 with a marginal *r*^2^ of 0.18 suggesting age, supplement intake, body weight and body condition accounted for 18% of the variation associated with distance traveled per day.

#### Resource utilization

**Fig 3:**
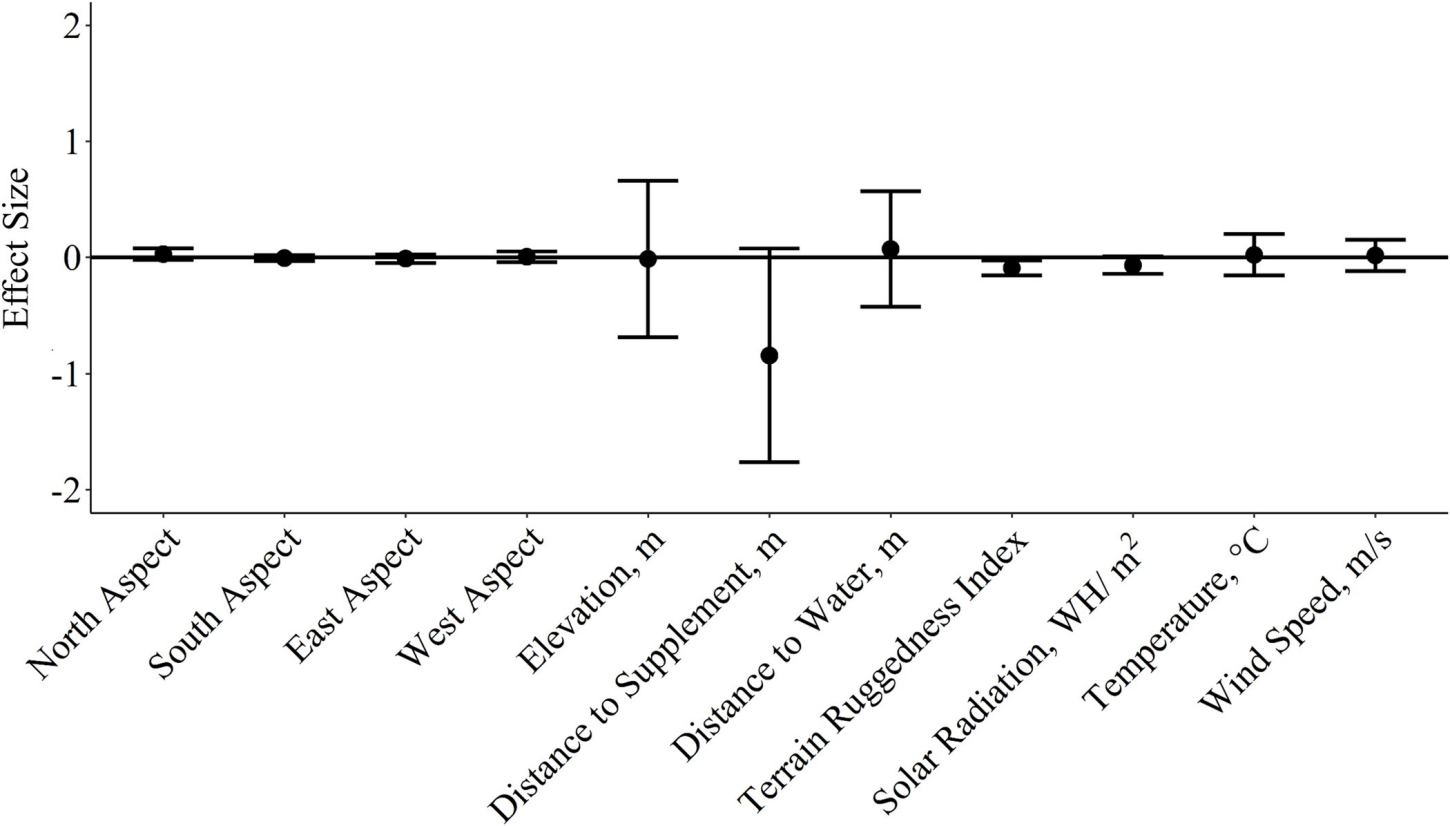
Mean standardized herd-level effect size ($\hat{\bar{\beta }}$ ± 95% CI) for cattle grazing resource utilization functions, 95% confidence intervals of $\hat{\bar{\beta }}$ that do not overlap zero denote significant responses at the population level.

We estimated RUFs for 58 cattle (29 per year) using an average of 910 ± 38 grazing locations per individual. Resource utilization by cattle grazing dormant season forage was negatively related to terrain ruggedness ($\hat{\bar{\beta }}$ = −0.09 ± 0.03; Fig 3). Additionally, relative selection by cattle tended to decrease with distance from supplement ($\hat{\bar{\beta }}$ = −0.84 ± 0.45); however, individual variability in selection resulted in confidence intervals overlapping 0 for the herd-level response (Fig 3). Aspect, elevation, distance from water, solar radiation, average temperature and wind speed had little influence on grazing space use at a population level within the study pasture. However, resource utilization relative to distance from supplement, distance from water, and elevation were highly variable among individuals with some individuals selecting for these conditions and others selecting against (herd level SE of standardized coefficients > 0.25; S1 Fig). Therefore, we conducted a post hoc analysis evaluating the relationship of individual cow attributes (age, body weight, body condition and average daily supplement intake) on resource utilization relative to distance to supplement, distance to water and elevation.

**Fig 4:**
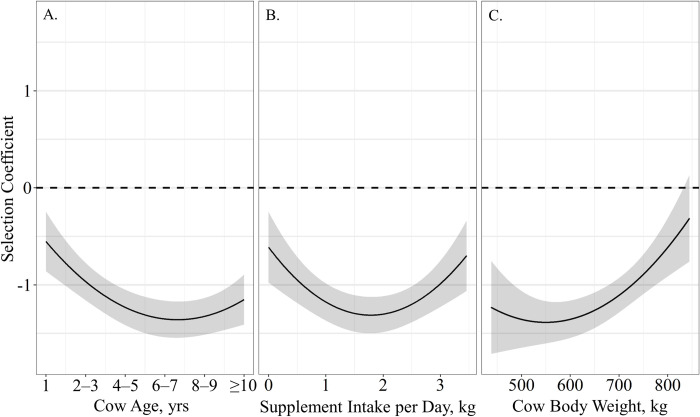
Predicted relationships (± 95%CI represented in the shaded area) between (**A**) cow age (years), (**B**) average supplement intake per day (kg) and (**C**) cow body weight (kg) on resource utilization relative to distance from supplement by cattle grazing dormant northern mixed grass rangeland in 2016 – 2017 & 2017 – 2018 at the Northern Agricultural Research Center Thackeray ranch, Havre, MT.

The probability of grazing site selection relative to distance from supplement was influenced by age, supplement intake and body weight, as a single top model containing age, supplement intake and body weight received 57% of the relative support among candidate models (Table 3). Resource utilization relative to distance from supplement was quadratically associated with cow age ($\hat{\beta }$_Age_ = −0.63 ± 0.15, $\hat{\beta }$_Age_^2^ = 0.07 ± 0.02; *P* < 0.01), indicating that cattle of all ages select for grazing locations close to supplement with cows 6 – 7 years of age being the most closely associated with supplement sites (Fig 4A). Likewise, resource utilization relative to distance to supplement had a quadratic relationship with supplement intake ($\hat{\beta }$_Supplement Intake_ = −0.78 ± 0.21, $\hat{\beta }$_Supplement Intake_^2^ = 0.22 ± 0.58; *P* < 0.01), where any supplement intake resulted in selection of grazing location near supplement feeders, with intakes ranging between 1.5 and 2-kg being the most closely associated with supplement sites (Fig 4B). Cow body weight also exhibited a quadratic effect on resource utilization relative to distance from supplement ($\hat{\beta }$_Weight_ = −0.01 ± 0.01, $\hat{\beta }$_Weight_^2^ = 0.001 ± 0.0001; *P* < 0.05), as cattle weight increased above 650-kg they became less associated with supplement location (Fig 4C).

**Table 3:** Model selection for models evaluating the effects of cow age, body condition and supplement intake on the effect size of distance from supplement, distance from water and elevation from the RUF analysis of cattle grazing dormant rangeland in 2016 – 2017 & 2017 – 2018 at the Northern Agricultural Research Center Thackeray ranch, Havre, MT.

Model^b^	K^c^	AICc^d^	ΔAICc^e^	*w*_*i*_^f^	*r*^2^^g^
Distance from Supplement					
Age^2^ + Supplement Intake^2^ + Body Weight^2^	8	60.74	0.00	0.57	0.45
Age^2^ + Supplement Intake^2^ + Body Weight^2^ + Body Condition^2^	10	63.01	2.27	0.18	0.48
Age^2^ + Body Condition^2^ + Age^2^ × Body Condition^2^	9	65.08	4.34	0.06	0.43
Constant (null)	2	80.21	19.47	0.00	
Distance from Water					
Age^2^ + ln(Supplement Intake) + Body Weight^2^ + Body Weight^2^ × ln(Supplement Intake)	9	-4.25	0.00	0.21	0.41
Age^2^ + ln(Supplement Intake) + Body Weight^2^	7	-3.52	0.73	0.15	0.34
ln(Supplement Intake) + Body Weight^2^ + Body Weight^2^ × ln(Supplement Intake)	7	-2.97	1.29	0.11	0.34
ln(Supplement Intake) + Body Weight^2^	5	-2.10	2.16	0.07	0.26
Age^2^ + ln(Supplement Intake)	5	-2.02	2.23	0.07	0.26
Age^2^ + Body Weight^2^	6	-1.84	2.41	0.06	0.29
Constant (null)	2	8.68	12.93	0.00	
Elevation					
Age Class + ln(Supplement Intake) + ln(Body Weight)	9	28.89	0.00	0.39	0.43
Age Class + ln(Supplement Intake) × ln(Body Weight)	7	29.66	0.77	0.27	0.36
Age Class + ln(Supplement Intake) + ln(Body Condition) + ln(Body Weight)	8	30.78	1.89	0.15	0.38
Constant (null)	2	43.69	14.80	0.00	

^a^Only models with Akaike weights (*w*_i_) ≥ 0.05 are presented except for the null model.

^b^Cow is used as a random variable in all models.

^c^K = number of parameters.

^d^Akaike’s information criterion adjusted for small sample size.

^e^Difference in Akaike’s information criterion adjusted for small sample size compared to the best model.

^f^Akaike weight.

^g^Multiple R^2^.

**Fig 5:**
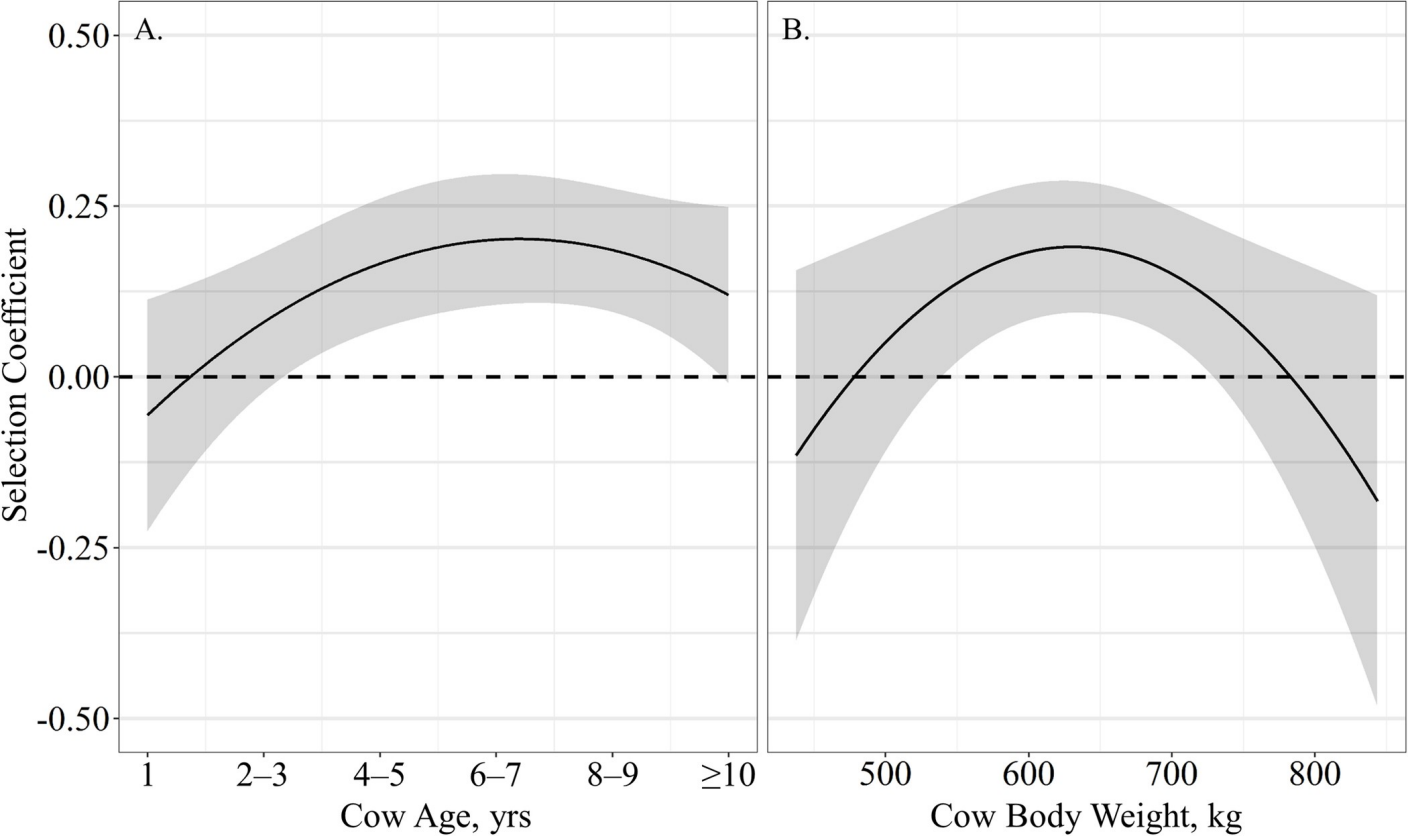
Predicted relationships (± 95%CI represented in the shaded area) between (**A**) cow age (years) and (**B**) cow body weight (kg) on resource utilization relative to distance from water by cattle grazing dormant northern mixed grass rangeland in 2016 – 2017 & 2017 – 2018 at the Northern Agricultural Research Center Thackeray ranch, Havre, MT.

The probability of grazing site selection relative to distance from water was also influenced by age, supplement intake and body weight, as models containing a quadratic effect of age and body weight, and an asymptotic effect of supplement intake and an interaction of supplement intake by body weight were most supported among candidate models (Table 3). However, supplement intake ($\hat{\beta }$ = 0.69 ± 1.97; *P* = 0.72) and the interaction of supplement intake by body weight ($\hat{\beta }$_Supplement Intake × Body Weight_ = −0.002 ± 0.007, $\hat{\beta }$_Supplement Intake × Body Weight_^2^ = 0.001 ± 0.001; *P* > 0.72) may be non-informative as confidence intervals of the effect size overlap 0. Resource use relative to distance from water was quadratically affected by cow age ($\hat{\beta }$_Age_ = 0.22 ± 0.09, $\hat{\beta }$_Age_^2^ = −0.03 ± 0.01; *P* < 0.03), where yearling to 3-yr-old cattle selected grazing locations regardless of proximity to water, while cattle 6 – 7 years of age selected grazing locations farthest from water (Fig 5A). Cow body weight also elicited a quadratic effect on resource use relative to distance from water ($\hat{\beta }$_Body Weight_ = 0.01 ± 0.005, $\hat{\beta }$_Body Weight_^2^ = −0.001 ± 0.0001; *P* < 0.02), as the lightest and heaviest cattle neither selected for or against distance to water and cattle weighing between 600 and 700 kg selected grazing locations away from water (Fig 5B).

**Fig 6:**
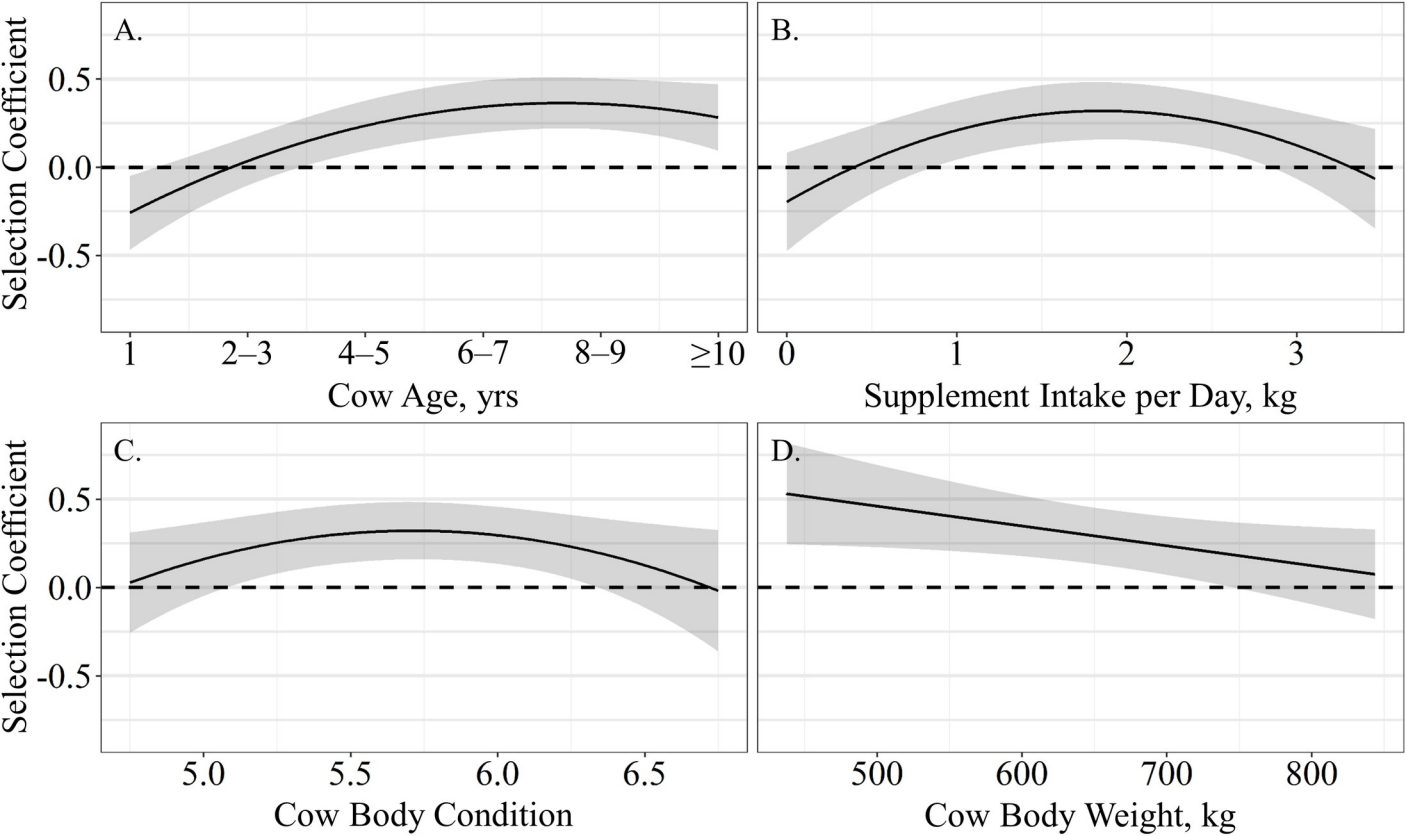
Predicted relationships (± 95%CI represented in the shaded area) between (**A**) cow age (years), (**B**) average supplement intake per day (kg), (**C**) cow body condition and (**D**) body weight (kg) on resource utilization relative to elevation by cattle grazing dormant northern mixed grass rangeland in 2016 – 2017 & 2017 – 2018 at the Northern Agricultural Research Center Thackeray ranch, Havre, MT.

The probability of grazing site selection relative to elevation was influenced by age and supplement intake as models containing a quadratic effect of age and supplement intake had 95% of the relative support among candidate models (Table 3). Models containing a linear effect of body weight and a quadratic effect of body condition were also supported. Resource use relative to elevation was quadratically associated with age ($\hat{\beta }$_Age_ = 0.41 ± 0.12, $\hat{\beta }$_Age_^2^ = −0.04 ± 0.02; *P* < 0.01), where yearlings selected grazing locations in lower elevations while older cattle selected grazing locations at higher elevations (Fig 6A). Resource use relative to elevation was also quadratically affected by supplement intake per day ($\hat{\beta }$_Supplement Intake_ = 0.56 ± 0.16, $\hat{\beta }$_Supplement Intake_^2^ = −0.15 ± 0.05; *P* < 0.01), where cattle consuming 0 or 3-kg of supplement per day selected grazing locations regardless of elevation, as animals that consumed approximately 1.5 kg of supplement per day utilized higher elevation areas for grazing (Fig 6B). Cow body condition also elicited a quadratic association on resource utilization relative to elevation ($\hat{\beta }$_Body Condition_ = 3.81 ± 1.55, $\hat{\beta }$_Body Condition_^2^ = −0.34 ± 0.14; *P* = 0.02), where cows with body condition of 5.5 – 6 selected areas at higher elevations to graze (Fig 6C). Resource use relative to elevation was negatively associated with body weight ($\hat{\beta }$= −0.001 ± 0.0005; *P* = 0.03), where lighter weight cattle selected grazing locations at higher elevations than heavier cattle (Fig 6D).

#### Vegetation chemical composition and production

**Fig 7:**
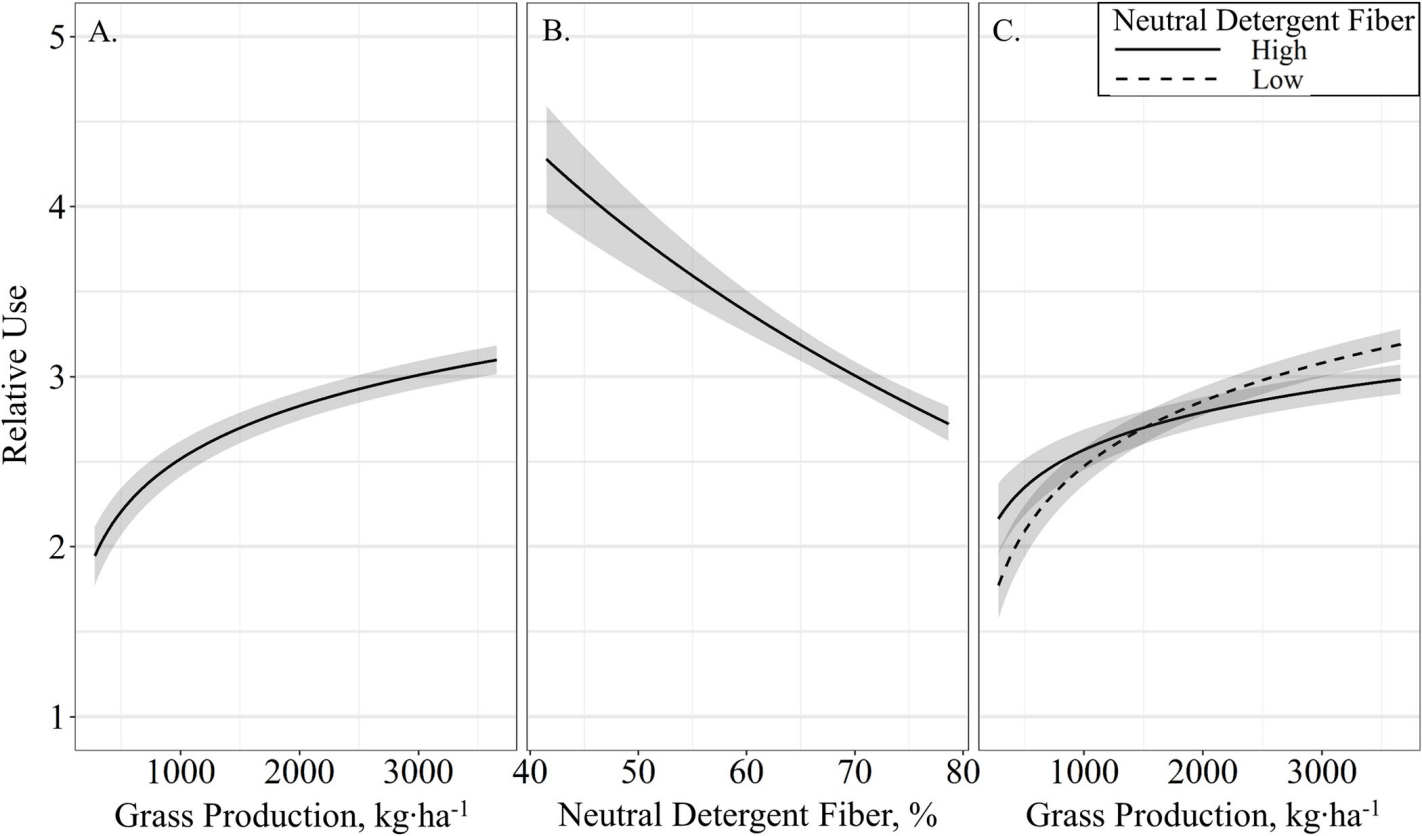
Predicted relationships (± 95%CI represented in the shaded area) between (**A**) grass production, (**B**), neutral detergent fiber, and (**C**), the interactions of grass production by neutral detergent fiber on relative resource utilization by cattle grazing dormant northern mixed grass rangeland in 2016 – 2017 & 2017 – 2018 at the Northern Agricultural Research Center Thackeray ranch, Havre, MT.

Relative use of fine-scale vegetation conditions was evaluated using the cumulative grazing densities of all cattle. The relationship of vegetation composition, production, and chemical composition were contrasted with cow age, body condition, body weight, average daily supplement intake and relative resource utilization by cattle. Models containing grass production (kg ∙ ha^-1^), neutral detergent fiber (%), and an interaction of grass production by neutral detergent fiber received virtually all the relative support among candidate models (Table 4). Models containing crude protein (%; $\hat{\beta }$ = 0.68 ± 1.67; *P* = 0.68) and an interaction of grass production by crude protein ($\hat{\beta }$ = −0.35 ± 0.18; *P* = 0.06) were also supported, though effects may be non-informative as confidence intervals of the effect size overlap 0. Relative use had an asymptotic relationship with grass production ($\hat{\beta }$ = 16.58 ± 3.25; *P* < 0.01), where predicted relative use increased non-linearly with grass production (Fig 7A). Neutral detergent fiber also displayed an asymptotic relationship with relative use ($\hat{\beta }$ = 27.19 ± 6.03; *P* < 0.01), where relative use decreased non-linearly with increasing NDF (Fig 7B). However, an interaction between NDF and grass production was supported ($\hat{\beta }$ = −3.71 ± 0.77; *P* < 0.01); high NDF values were selected in areas of low grass production, while low NDF values were selected in areas of high grass production (Fig 7C).

**Table 4:** Model selection for models evaluating the effects of vegetation chemical composition and production, cow age, body condition, body weight and supplement intake on grazing resource utilization by cattle grazing dormant rangeland in 2016 – 2017 & 2017 – 2018 at the Northern Agricultural Research Center Thackeray ranch, Havre, MT.

Model^b^	K^c^	AICc^d^	ΔAICc^e^	*w*_*i*_^f^	*r*^2^m^g^	*r*^2^c^h^
ln(Grass Production) × ln(Neutral Detergent Fiber) + ln(Grass Production) × ln(Crude Protein)	8	12369.22	0.00	0.51	0.05	0.13
ln(Grass Production) × ln(Neutral Detergent Fiber) + ln(Crude Protein)	7	12369.31	0.07	0.49	0.05	0.13
Constant (null)	3	12582.92	213.68	0.00		

^a^Only models with Akaike weights (*w*_i_) ≥ 0.05 are presented except for the null model.

^b^Cow is used as a random variable in all models.

^c^K = number of parameters.

^d^Akaike’s information criterion adjusted for small sample size.

^e^Difference in Akaike’s information criterion adjusted for small sample size compared to the best model.

^f^Akaike weight.

^g^Marginal R^2^.

^h^Conditional R^2^.

### Vegetation structure and heterogeneity

#### Structure

**Fig 8:**
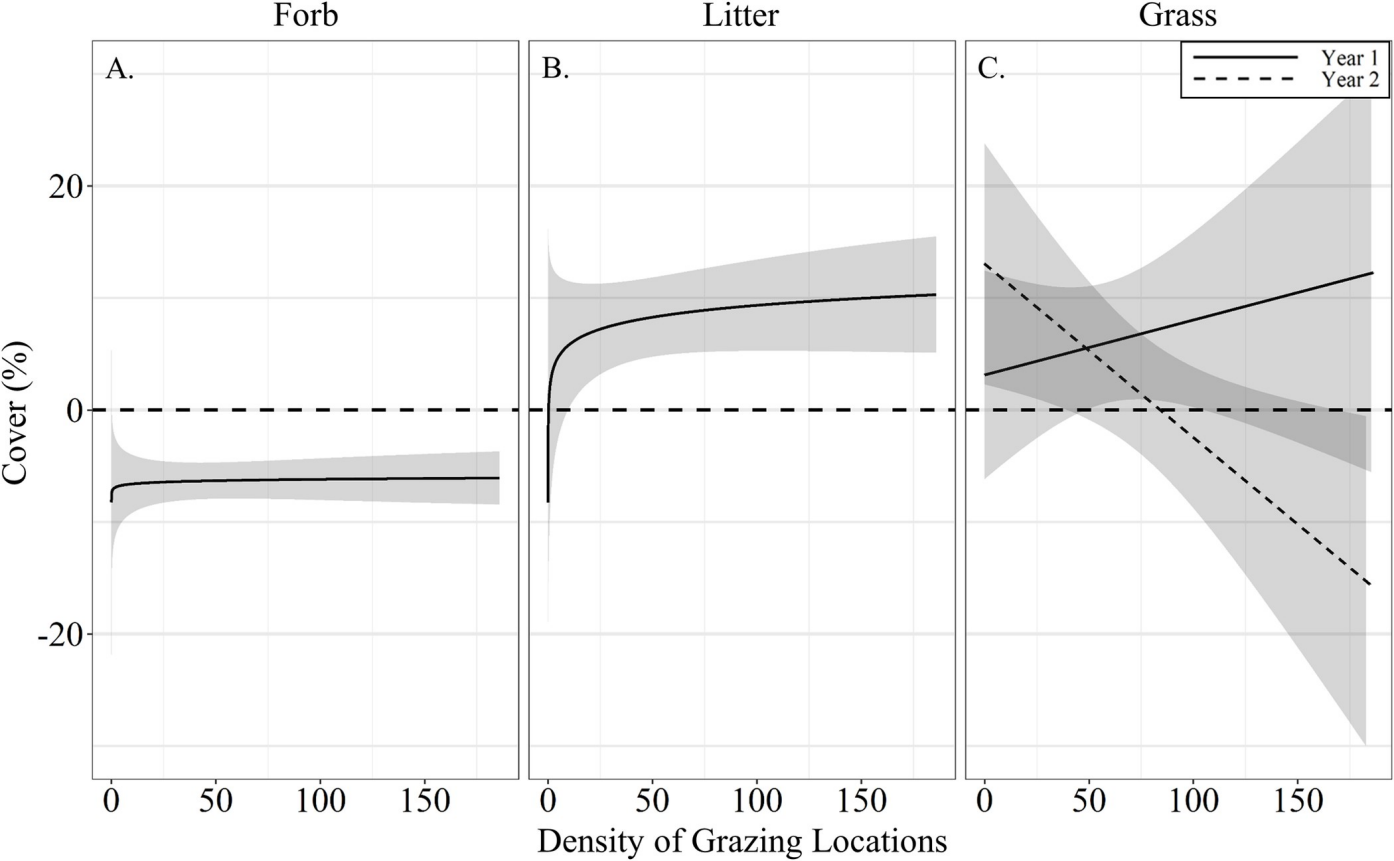
Predicted relationships (± 95%CI represented in the shaded area) of pre- post grazing differences of residual cover of (**A**) forb, (**B**) litter, (**C**) a grass by year interaction and the density of grazing locations within a 50 m radius of transect locations by cattle grazing dormant northern mixed grass rangeland in 2016 – 2017 & 2017 – 2018 at the Northern Agricultural Research Center Thackeray ranch, Havre, MT.

Livestock grazing did not affect pre-post differences in mean VOR ($\hat{\beta }$ = 0.001 ± 0.012; *P* = 0.25), bare ground cover ($\hat{\beta }$ = 0.003 ± 0.016%; *P* = 0.64) and residual cover of grass ($\hat{\beta }$ = 0.05 ± 0.07%; *P* = 0.22; Table 5). However, the data supported a positive asymptotic relationship between residual cover of forbs and grazing intensity ($\hat{\beta }$ = 1.04 ± 0.41%; *P* = 0.05), where residual forb cover was reduced at all densities of grazing locations but displayed a non-linear increase in cover with density of grazing locations (Fig 8A). Ground cover of litter also had a positive asymptotic relationship with density of grazing locations ($\hat{\beta }$ = 3.06 ± 0.89%; *P* < 0.01), where increasing density of grazing locations resulted in a non-linear increase of litter ground cover (Fig 8B). A year by density of grazing locations (grazing intensity) interaction in residual grass cover was also supported ($\hat{\beta }$ = −0.20 ± 0.09%; *P* = 0.03), where in year one grazing intensity had a slight positive effect on residual grass cover, while in year 2 grass cover was negatively associated with grazing intensity (Fig 8C). Visual obstruction reading, bare ground and litter cover all displayed a significant year effect (*P* < 0.01; Table 5). Bare ground decreased by 1.41 ± 0.43% after grazing in year one with no difference in year two (1.32 ± 0.79%; Table 5). Conversely, litter cover increased 14.19 ± 2.22% in year one with no differences in year two (0.91 ± 1.33%; Table 5). Visual obstruction was decreased in both years, however, had a larger reduction in year two than year one (-7.96 ± 0.62 vs -5.94 ± 0.29; Table 5).

**Table 5:** Pre- post differences in mean (± SE) visual obstruction and residual cover classifications by year for cattle grazing dormant rangeland in 2016 – 2017 & 2017 – 2018 at the Northern Agricultural Research Center Thackeray ranch, Havre, MT.

			P-Value		
	Year 1	Year 2	Year	Grazing Intensity	Year × Grazing Intensity
**VOR**^a^**, cm**	-5.94 ± 0.29	-7.96 ± 0.62	< 0.01	0.25	0.23
**Bare Ground, %**	-1.41 ± 0.43	1.32 ± 0.79	< 0.01	0.64	0.49
**Litter, %**	14.19 ± 2.22	0.91 ± 1.33	< 0.01	< 0.01	0.15
**Grass, %**	5.95 ± 2.60	1.58 ± 2.96	0.26	0.22	0.03
**Forb, %**	-6.42 ± 0.86	-6.78 ± 0.79	0.76	0.05	0.08

^a^Visual Obstruction Reading.

#### Heterogeneity

**Fig 9:**
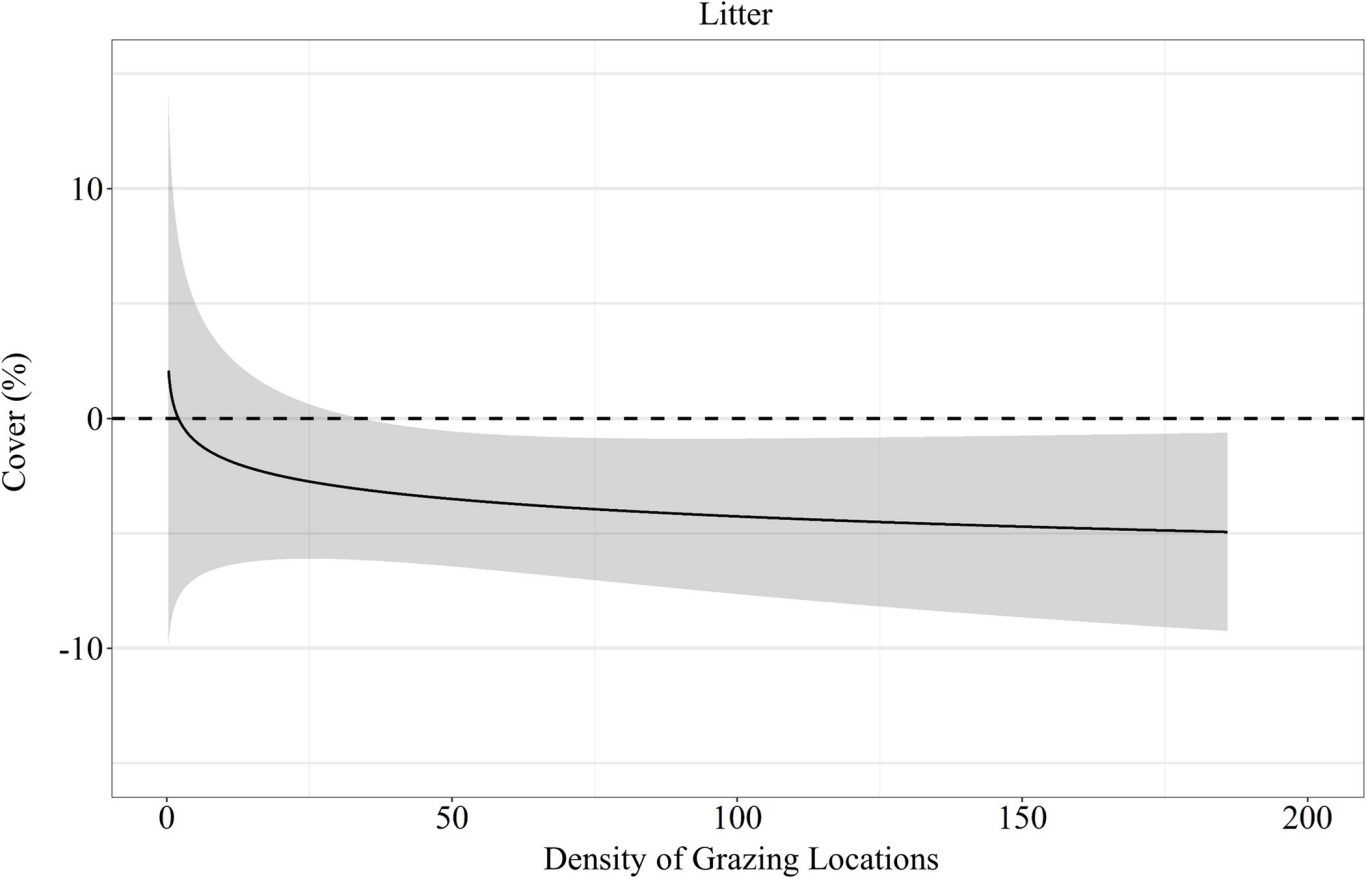
Predicted relationships (± 95%CI represented in the shaded area) between pre- post grazing heterogeneity (as indexed by difference of standard deviation among transects) of litter and density of grazing locations within a 50 m radius of transect by cattle grazing dormant northern mixed grass rangeland in 2016 – 2017 & 2017 – 2018 at the Northern Agricultural Research Center Thackeray ranch, Havre, MT.

Grazing intensity did not significantly alter the differences in transect level standard deviation of VOR ($\hat{\beta }$ = 0.009 ± 0.009; *P* = 0.63), bare ground ($\hat{\beta }$ = −0.001 ± 0.021%; *P* = 0.63), or residual grass ($\hat{\beta }$ = −0.02 ± 0.03%; *P* = 0.41) and forb cover ($\hat{\beta }$ = 0.54 ± 0.39%; *P* = 0.36; Table 6). Difference in pre- post grazing standard deviation of litter had an asymptotic relationship with density of grazing locations ($\hat{\beta }$ = 2.21 ± 0.74%; *P* < 0.01), whereas slight increasing density of grazing locations results in a reduction in the pasture level standard deviation of litter (Fig 9). Visual obstruction reading and litter cover difference in pre- post grazing standard deviation displayed a significant year effect (*P* < 0.02; Table 6). Visual obstruction standard deviation was decreased in both years, with a larger reduction in transect standard deviation in year two than year one (-3.21 ± 0.46 vs -1.90 ± 0.27; Table 6). Pre- post grazing difference of litter standard deviation was decreased 5.36 ± 1.50% in year one with no differences in year two (−0.57 ± 1.52%; Table 6).

**Table 6:** Pre-post difference in heterogeneity (as indexed by differences in standard deviation ± SE) of visual obstruction and residual cover classifications by year for cattle grazing dormant rangeland in 2016 – 2017 & 2017 – 2018 at the Northern Agricultural Research Center Thackeray ranch, Havre, MT.

			P-Value		
	Year 1	Year 2	Year	Grazing Intensity	Year × Grazing Intensity
**VOR**^a^**, cm**	-1.90 ± 0.27	-3.21 ± 0.46	0.02	0.63	0.36
**Bare Ground, %**	-0.64 ± 0.57	1.53 ± 1.08	0.08	0.63	0.71
**Litter, %**	-5.36 ± 1.50	-0.57 ± 1.52	0.02	0.01	0.20
**Grass, %**	-0.77 ± 1.31	0.76 ± 1.28	0.41	0.41	0.74
**Forb, %**	-5.03 ± 0.71	-5.72 ± 0.84	0.54	0.36	0.20

^a^Visual Obstruction Reading.

## Discussion

Cow age, body condition, weight and supplement intake are known to have substantial effects on intake of low-quality forage [5, 17, 65]. Our research suggests these individual level factors also have considerable effects on grazing behavior and resource use in relation to landscape variables (e.g. elevation and distance from supplement and water) that result in high amounts of herd-level variability. The cow herd in our study grazes the same pasture each winter, thus, cow age also reflects experience. Our results are consistent with previous research that has demonstrated experienced cattle are more likely to use areas farther from water and higher in elevation [15, 66]. Additionally, our research suggests that older cattle graze closer to supplement locations. Therefore, supplement location and previous experience likely interact in determining cattle grazing locations. Time spent grazing was greatest for mid-aged cows (4 – 7 years-old). Previous research has established that older cows spend more time grazing than younger cows [19], however, categorical age treatments allowed inference for only 3- and 6-year-old cattle. The difference in time spent grazing per day is likely due to inexperience of younger cattle grazing dormant rangelands [5] and a decrease in structural soundness and production efficiency of cattle > 8 years of age [67].

Providing supplement to cattle grazing dormant forage often results in decreased time spent grazing [5, 68]. This negative association suggests that as cattle increase supplement intake, they either decrease forage intake or increase grazing intensity and harvest efficiency. Cattle alter their grazing behavior in response to nutritional factors, where providing a protein supplement while grazing dormant forage can increase grazing intensity, harvest efficiency and forage intake with an overall decrease in total time spent grazing [5, 27]. However, cattle have been shown to decrease their intake of low-quality forage if supplement is consumed greater than 0.8% of body weight [65]. The average weight of cattle across both years of the study was 627 kg, suggesting that any daily supplement intake over 5 kg would depress forage intake, likely reflected in time spent grazing. In addition, NDF has been proposed as the most important factor influencing forage intake of ruminants, where a positive response of protein supplementation on forage intake would only be expected when NDF intake is less than 12.5-g ∙ kg of body weight^-1^ ∙ d^-1^ [69, 70]. During both years of our study, the available forage base averaged 70% NDF and less than 7% CP, indicating that forage was not adequate in meeting the nutrient requirements of a non-lactating beef cow. The NDF values at the initiation of the grazing study likely maintained or increased slightly due to the winter environment as both years’ average temperatures during the grazing period were below 0°C. Thus, if cattle were to consume a minimum 11.2 kg of forage per day the positive effects of protein supplementation on forage intake would likely be negated. At the stocking rate used in our study, overall forage use was estimated at less than 30% of total available forage at study initiation for both years, suggesting that forage availability did not impact forage intake during the later portions of the winter grazing period. Therefore, it is probable that as cattle in our study increased supplement intake, they decreased forage intake and subsequently total time spent grazing.

Traveling is believed to influence the energy requirements of grazing livestock [71]. However, previous studies show mixed results on the effects of supplementation on distance traveled [21, 27, 68]. The majority of these studies specifically looked at the effects of supplemented vs non-supplemented cattle and did not measure the relationship between individual animal supplement intake and distance traveled. In our study, cattle increased travel rapidly with increased supplement intake until animals consumed approximately 2.0-kg per day at which a distance traveled per day met a semi-threshold. The energetic costs associated with travel can increase maintenance requirements from 10 – 25% in grazing animals [72, 73]. Therefore, cattle in our study may have increased consumption of supplement in response to increased energy expenditure associated with traveling to the point supplement intake may limit foraging activity and consequentially distance traveled.

Additionally, we found evidence that cow body condition at the initiation of the study mediated the effect of supplement intake on distance traveled. Generally, cows consuming low levels of supplement traveled relatively little, however, cattle with relatively low body condition (< 5) traveled farther per unit increase of supplement intake than cattle with high body condition (> 6). Livestock in relatively low body condition typically increase forage intake [17, 74, 75]. Although our study did not measure forage intake, our data suggests cattle may be consuming supplement as a substitute for dormant forage. Therefore, it may be reasonable to assume a similar relationship between cattle body condition and supplement intake as cattle with relatively low body condition consumed more supplement than cattle with high body condition (2.03 ± 0.06, 1.14 ± 0.17 kg). Thus, the interaction of supplement intake and body condition may reflect the nutrient status of the animal in relation to energy expenditure associated with travel.

Contrary to expectations, elevation and distance from water and supplement were not significant drivers in herd level resource use in our study. However, these parameters exhibited substantial amounts of individual variation, suggesting cattle resource use relative to elevation and distance from water and supplement is influenced by individual animal attributes. Individual average daily supplement intake, body weight, and condition were all important factors in determining the extent of selection of grazing locations relative to elevation and distance from supplement and water. The effects of individual animal attributes on resource use are likely related to energetic requirement for maintenance. Energetic requirements for maintenance are directly related to the metabolic body weight of the animal (BW^0.75^), with activity increasing energy requirements per unit body weight [3]. Thus, the energetic cost of traveling to higher elevations is increased for heavier weight cattle. This may explain why heavier cattle in our study had a lower selection for grazing locations close to supplement and higher elevations away from water. In general, supplement intake may mitigate the increased energetic cost of travel to higher elevation grazing locations for cattle. However, in our study as average daily supplement intake increased the selection of grazing locations in higher elevations only increased until animals consumed approximately 2-kg of supplement per day, after which selection for elevation decreased. High levels of supplement intake may result in cattle consuming supplement as a substitute to forage, decreasing overall forage intake and time spent grazing, resulting in the changes in resource use relative to elevation and distance from supplement.

Our results are consistent with previous work that have demonstrated cattle avoid rough terrain [12, 15, 76] and select grazing locations in areas with relatively high forage production and quality [15, 77, 78]. Our results support an interaction between grass production and NDF, where cattle selected grazing locations with high levels of NDF in areas of low grass production while selecting areas of low NDF in areas of high grass production. At our study site, low grass production areas were typically dominated by C-4 grasses (e.g. *Schizachyrium scoparium*) or bunch grasses with a low leaf:stem ratio (e.g. *Pseudoroegneria spicata*), both of which were relatively high in NDF. Therefore, we attribute the interaction of grass production and NDF on cattle resource utilization to low production areas being inherently higher in NDF.

Grazing intensity assessed through density of grazing locations had a significant effect on forb and litter cover. Virtually any grazing resulted in an increase in forb cover that rapidly met a threshold. Cattle typically have a strong dietary preference for grasses [79–81]. Thus, increases in forb cover with grazing intensity is likely an artifact of cattle removing grass biomass via grazing, increasing forb detection during the post-grazing vegetation data collection period. Grazing intensity also increased litter cover and reduced heterogeneity of litter cover, both of which rapidly met thresholds. Previous research evaluating the effects of grazing on litter suggests that dormant standing vegetation is trampled, broken into smaller pieces, and categorized as litter post-grazing [82]. Therefore, grazing intensity would be expected to increase litter cover to the point at where vegetation removal by grazing limits litter accumulations. Additionally, increases in litter cover may reduce over all heterogeneity of litter cover.

Year had a significant effect on bare ground and litter cover, VOR, and the effect of grazing intensity on grass cover, presumably due to differences in weather between the two years of the grazing trial. On average, the first year of our trial was 7.6°C colder than year two. Although year two received higher amounts of total precipitation, colder temperatures in year one resulted in an increased snowfall earlier in the grazing season and prolonged time periods of snow ground cover. The second year of the study also received snowfall early in the grazing period, however, warm temperatures limited prolonged periods of snow ground cover until late in the trial. Snow cover limits forage availability of grazing cattle [78, 83] and can have major effects on grazing behavior. The availability of forage forms the bounds from which the animal selects its diet and high forage availability allows cattle to graze selectively [84, 85]. Limited forage availability likely caused animals to consume a greater proportion of the less-preferred forage [84], and focus grazing efforts in areas where less snow had accumulated. Our data supports this behavioral response as grazing intensity had little to no effect on grass cover in year one, even though cattle have strong dietary preference for grass [79–81]. However, the second year of the grazing trial resulted in a strong negative association between grass cover and grazing intensity. Limited forage availabilty is further evident as the first year of the study resulted in an increase in litter with a subsequent decrease litter heterogeneity and bare ground, while in the second year neither were changed. Snow covered vegetation unavailable for grazing would likely result in an increase in litter cover and homogenity post grazing. Despite the potential interaction of grazing behavior and forage availability due snow cover, at the stocking rate used in our study, overall forage use was estimated at approximately 30% of total available forage at study initiation. Visual obstruction readings and heterogeneity displayed greater decreases the second year than in year one of the grazing trial, however, it is unclear if these findings are due to forage availability in relation to snow cover or the fact that the grazing season in the second year was approximately 2 weeks longer than the first year.

### Implications

We observed high individual variability in grazing site selection of cattle, suggesting individual-level factors could be the dominant drivers in grazing resource use and behavior. Our research shows that at low to moderate stocking rates where forage is not limiting the combination of age, supplement intake, weight and body condition, can interact with the environmental attributes of the landscape to influence grazing behavior resulting in significant implications in animal and land management. Cattle experience, nutrient status and the energetic cost of grazing activity may be dominant drivers in cattle resource utilization. Individual variation in supplement intake has the potential to influence individual animal nutrient status and performance, thus altering grazing behavior and use of forage resources across a pasture. Monitoring daily grazing behavior without accounting for individual level factors may not provide meaningful insight about the complex interrelationships that exist between grazing livestock and their environment. Future research examining the effects of supplementation on grazing behavior and resource use should incorporate individual animal measurements in an attempt to account for individual animal variablity. Incorporating measurements of animal performance, forage intake and energetic costs associated with travel and grazing activities could provide meaningful insight to the mechanisms driving grazing behavior and distribution. Understanding the effects of supplementation and variation in supplement intake on animal performance, behavior and paddock use are essential in the development of a cost effective and sustainable supplementation program for dormant season grazing.

Our research suggests that at low to moderate stocking rates for cattle winter grazing dormant forages, cattle select grazing locations based on the relative quantity and NDF content of available forage. Additionally, supplement intake can have an effect on the distance traveled, total time spent grazing per day and grazing resource use. For landscape attributes with substantial variablity in herd-level resource use, individual-level measurements (body weight and condition, age, supplement intake) were found to be significant predictors cattle resource use. Grazing intensity had little effect on vegetation conditons and spatial variability, however, this may be related to pasture vegetation and weather conditions at the time of grazing. The effect of winter grazing on vegetative conditions, as well as, spatial variability of vegetation (ie. heterogeneity) appeared to be strongly influenced by the weather conditions. Prolonged periods of snow cover presumably limited forage availability, reducing grazing selection and the overall effects of grazing on vegetation conditions.

## Supporting information

S1 Table Average winter temperature (low, high, mean; °C) and total precipitation (cm) for the 2 years of grazing (2016 – 2017, 2017 – 2018) at the Northern Agricultural Research Center Thackeray ranch, Havre, MT. (PDF)

S2 Table Supplement composition for cattle grazing dormant rangeland in 2016 – 2017 & 2017 – 2018 at the Northern Agricultural Research Center Thackeray ranch, Havre, MT (as-fed basis). (PDF)

S3 Table Cow weight (unshrunk) and body condition by age class for cattle grazing dormant rangeland in 2016 – 2017 & 2017 – 2018 at the Northern Agricultural Research Center Thackeray ranch, Havre, MT. (PDF)

S1 Fig Percent of GPS collared cattle with positive, neutral, and negative selection coefficients relative to elevation, distance to supplement and distance to water from the resource utilization analysis for cattle grazing dormant northern mixed grass rangeland in 2016 – 2017 & 2017 – 2018 at the Northern Agricultural Research Center Thackeray ranch, Havre, MT. (TIF)

S1 Appendix (CSV)

S2 Appendix (CSV)

S3 Appendix (CSV)

S4 Appendix (CSV)

S5 Appendix (CSV)

S6 Appendix (CSV)
