# Supplementary material for: Dormant season grazing on northern mixed grass prairie agroecosystems: Does protein supplement intake, cow age, weight and body condition impact beef cattle resource use and residual vegetation cover?
Source: PLoS One. 2020 Oct 13;15(10):e0240629. doi: 10.1371/journal.pone.0240629 (PMC7553296; doi:10.1371/journal.pone.0240629)
Supplement: S1 Table — (PDF) [file pone.0240629.s001.pdf]

S1 Table. Average winter temperature (low, high, mean; °C) and total precipitation (cm) for the 2 years of grazing (2016 – 2017, 2017 – 2018) at the Northern Agricultural Research Center Thackeray ranch, Havre, MT

	Temperature, °C			
	**Low**	**High**	**Mean**	**Precipitation (cm)**
**Year 1**	-22.70	3.80	-9.60	2.90
**Year 2**	-24.30	14.90	-2.00	4.06
