# Supplementary material for: Dormant season grazing on northern mixed grass prairie agroecosystems: Does protein supplement intake, cow age, weight and body condition impact beef cattle resource use and residual vegetation cover?
Source: PLoS One. 2020 Oct 13;15(10):e0240629. doi: 10.1371/journal.pone.0240629 (PMC7553296; doi:10.1371/journal.pone.0240629)
Supplement: S2 Table — (PDF) [file pone.0240629.s002.pdf]

S2 Table. Supplement composition for cattle grazing dormant rangeland in 2016 – 2017 & 2017 – 2018 at the Northern Agricultural Research Center Thackeray ranch, Havre, MT (as-fed basis)

**CP[1]**	30.00 %
**Crude fat**	1.00 %
**Crude fiber**	8.00 %
**Ca**	2.00 %
**P**	1.00 %
**Salt**	25.00 %
**K**	0.75 %
**Se**	1.50 ppm
**Vitamin A**	9,072.00 IU · kg^{-1}
**Vitamin D**	907.00 IU · kg^{-1}
**Vitamin E**	9.00 IU · kg^{-1}

[1]9.9% non-protein N
