# Supplementary material for: Dormant season grazing on northern mixed grass prairie agroecosystems: Does protein supplement intake, cow age, weight and body condition impact beef cattle resource use and residual vegetation cover?
Source: PLoS One. 2020 Oct 13;15(10):e0240629. doi: 10.1371/journal.pone.0240629 (PMC7553296; doi:10.1371/journal.pone.0240629)
Supplement: S3 Table — (PDF) [file pone.0240629.s003.pdf]

S3 Table. Cow weight (unshrunk) and body condition by age class for cattle grazing dormant rangeland in 2016 – 2017 & 2017 – 2018 at the Northern Agricultural Research Center Thackeray ranch, Havre, MT

	Age									
	**1**	**2**	**3**	**4**	**5**	**6**	**7**	**8**	**9**	**≥ 10**
**2016 – 2017**										
**Initial**										
**Body Weight, kg**	559.4	584.4	656.7	682.9	688.2	706.8	711.4	697.9	745.4	668.8
**Body Condition**	5.35	5.13	5.31	5.65	5.62	5.66	6.1	6.0	6.1	5.7
**Final**										
**Body Weight, kg**	510.7	546.2	608.2	635.1	637.6	653.0	668.5	667.0	682.7	630.9
**Body Condition**	5.55	5.30	5.46	5.80	5.60	5.81	5.9	6.0	5.5	5.6
**2017 – 2018**										
**Initial**										
**Body Weight, kg**	497.1	545.5	579.6	623.2	656.3	644.4	647.6	660.0	673.6	660.0
**Body Condition**	5.88	5.27	5.37	5.42	5.65	5.44	5.5	5.5	5.5	5.2
**Final**										
**Body Weight, kg**	493.2	543.1	566.3	598.6	626.6	611.8	616.9	628.2	645.2	604.4
**Body Condition**	5.57	5.30	5.16	5.19	5.42	5.26	5.4	5.3	5.3	4.9
