# Supplementary figures and images for: Dormant season grazing on northern mixed grass prairie agroecosystems: Does protein supplement intake, cow age, weight and body condition impact beef cattle resource use and residual vegetation cover?
Source: PLoS One. 2020 Oct 13;15(10):e0240629. doi: 10.1371/journal.pone.0240629 (PMC7553296; doi:10.1371/journal.pone.0240629)

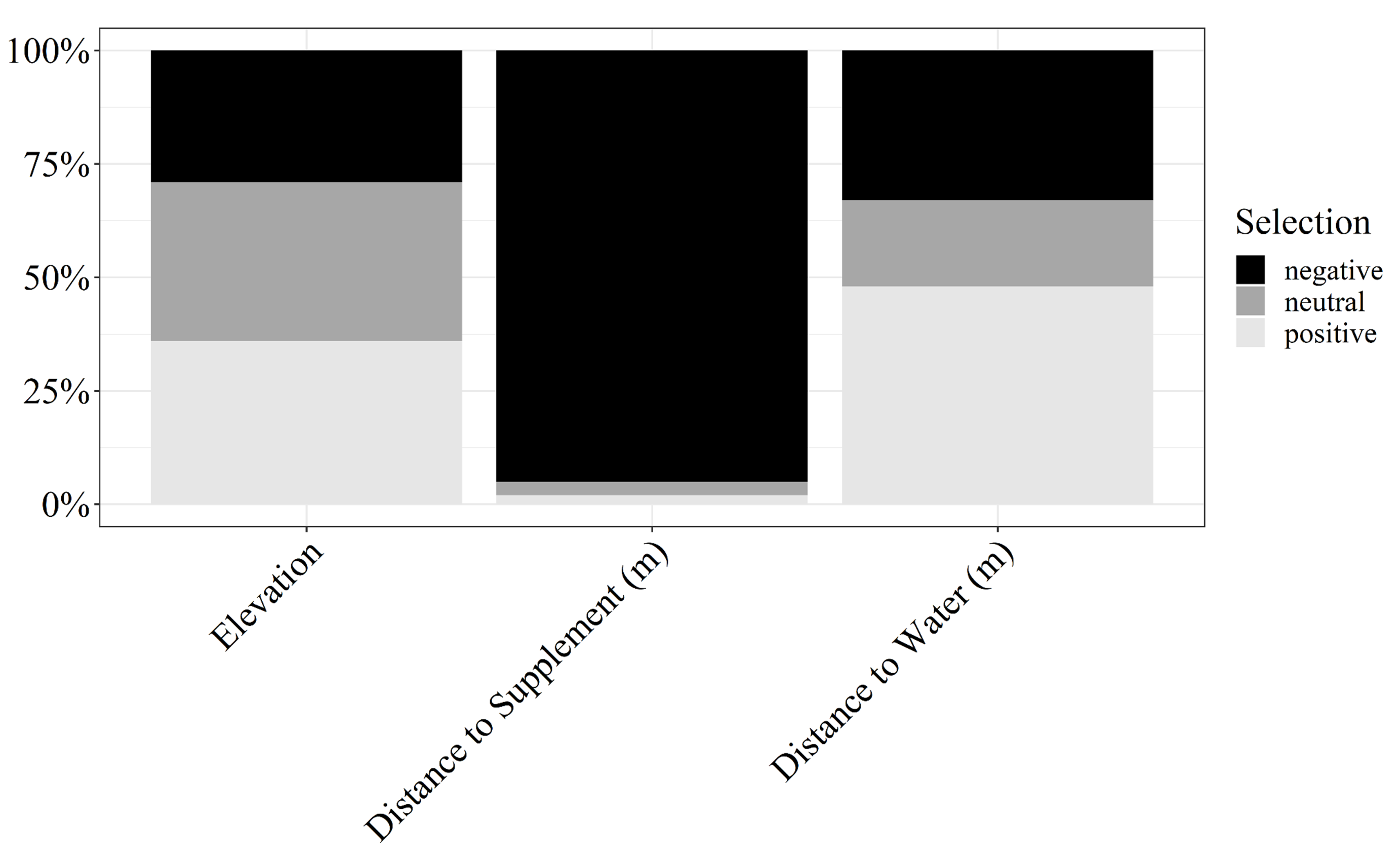

Supplement: S1 Fig — (TIF) [file pone.0240629.s004.tif]
